# Successful retrieval of lower limbs artery bone cement embolization resulting from percutaneous vertebroplasty: A rare case report

**DOI:** 10.1016/j.heliyon.2024.e41463

**Published:** 2024-12-25

**Authors:** Jiashen Shao, Hai Feng, Bin Liu, Hai Meng, Shili Ning, Yingchi Yang, Yun Yang, Xuehu Xie, Zihan Fan, Zhiwu Zhang, Nan Su, Jinjun Li, Qi Fei

**Affiliations:** a Department of Orthopedics, Beijing Friendship Hospital, Capital Medical University, Beijing, 100050, China; b Department of Vascular Surgery, Beijing Friendship Hospital, Capital Medical University, Beijing, 100050, China; c Department of General Surgery, Beijing Friendship Hospital, Capital Medical University, Beijing, 100050, China

**Keywords:** Percutaneous vertebroplasty, Pathologic vertebral fractures, Arterial embolization, Bone cement leakage

## Abstract

Percutaneous vertebroplasty (PVP) is a widely utilized minimally invasive technique originally developed for the treatment of vertebral compression fractures. It has since expanded to treat osteoporotic vertebral compression fractures, pathologic vertebral fractures resulting from primary or secondary spinal tumors, and traumatic spinal fractures. Despite its benefits, PVP is associated with significant complications, the most common of which is bone cement leakage. Arterial embolization due to cement leakage is a rare but increasingly recognized complication of PVP. Previous reports have documented cases of cement migrating into the aorta, renal arteries, and lower extremity arteries. However, with the growing use of PVP, the incidence of such vascular complications may rise. In this report, we present a rare case of bone cement leakage through the vertebral artery, leading to embolization in the inferior mesenteric artery and lower extremity arteries. The arterial embolus in the lower extremity was successfully treated with arteriotomy, highlighting the severe potential consequences of this complication and the importance of prompt recognition and intervention.

## 1 Introduction

Percutaneous vertebroplasty (PVP), which was originally developed as a minimally invasive technique for the treatment of vertebral compression fractures, is now widely used for the treatment of osteoporotic vertebral compression fractures, pathologic vertebral fractures due to primary or secondary spinal tumors, and traumatic spinal fractures [[1], [2], [3]]. Though PVP is a relatively minimally invasive and safe technique, it is still accompanied by a significant complication rate. One of the most common complications is cement leakage, which may occur at a reported rate of up to 73 % [4,5]. Bone cement may leak into the epidural, intradiscal, intervertebral foramina, paravertebral and even arteriovenous systems. Although most of these complications are asymptomatic, they may still lead to severe consequences.

Arterial embolization due to cement leakage and migration during PVP has been described rarely in previous reports [6,7]. However, with the widespread use of percutaneous vertebroplasty techniques, the incidence of this complication may increase. Leakage of bone cement into the arterial system, including leakage into the aorta [8], renal arteries [6], and lower extremity arteries [9], has also been reported in several previous studies. Here, we report a case of leakage of bone cement through the vertebral artery into the inferior mesenteric artery and lower extremity arteries with severe consequences.

## 2 Case report

**Fig. 1:**
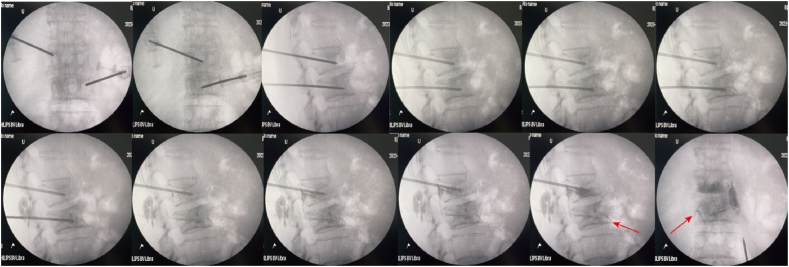
The process of injecting bone cement into the L2 and L3 vertebrae. The high-density material can be observed to leak out of the left anterior aspect of the L3 vertebrae in a tubular shape (red arrow).

A 70-year-old female patient was admitted to the hospital with persistent, severe back pain of unknown etiology that had persisted for two weeks, unresponsive to conservative treatment. Her medical history is significant for a left-sided breast carcinoma treated with a modified radical mastectomy a decade ago. Following surgery, she received adjuvant chemotherapy and targeted therapy, with no evidence of recurrence or metastasis during a two-year follow-up period. However, after this two-year follow-up, no further breast cancer-related surveillance or treatment was performed. Two weeks prior to the current presentation, the patient developed back pain, prompting further evaluation with PET/CT imaging. The imaging findings demonstrated multiple areas of osseous abnormalities with increased metabolic activity involving the left scapula, vertebral bodies, and bilateral iliac bones. The L2 and L3 vertebral bodies showed mild compression and slight concavity of the superior endplates, while a low-density osteolytic lesion was noted in the L4 vertebral body. Given the patient's clinical history, these findings are highly suggestive of extensive bone metastases secondary to breast cancer. Apart from her history of breast cancer, the patient has no other significant comorbid conditions. PVP was performed under local anesthesia in the operating room. The patient was placed in the prone position, and PVP was performed with two 13-gauge needles through bilateral pedicle approaches. Prepared bone cement was injected into the diseased vertebral bodies under fluoroscopic guidance. injections into the L2 and L4 vertebrae went smoothly. However, when the cement was injected into the L3 vertebral body, there was a delay in the visualization of the cement that did not correspond to the amount injected. It was found that some of the cement leaked out of the pedicle into the subcutaneous area. At this time, the patient complained of left lower extremity pain and abdominal pain. We immediately stopped the injection in this vertebra. Intraoperative fluoroscopy was performed at the end of the procedure, and the cement in the L3 vertebral body was found to have spilled into the blood vessels (Fig. 1).

**Fig. 2:**
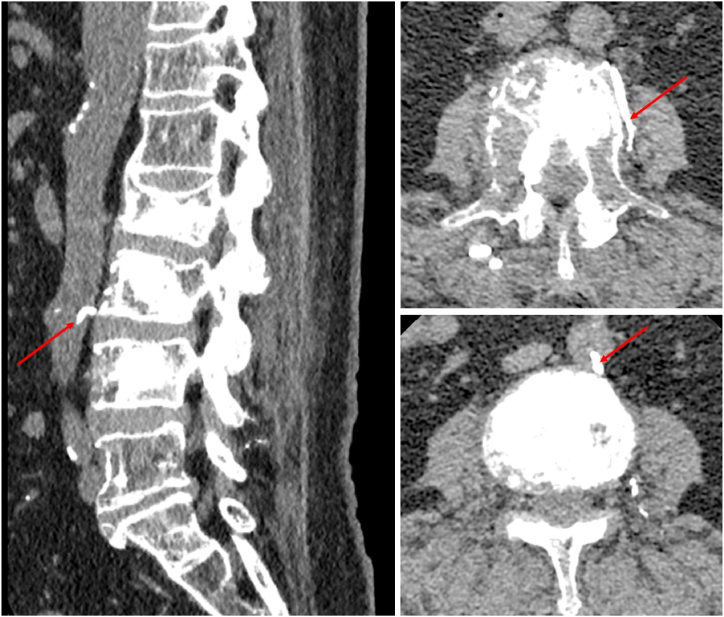
Postoperative lumbar CT sagittal and transverse views showing linear leakage of the third lumbar segmental artery (red arrows).

**Fig. 3:**
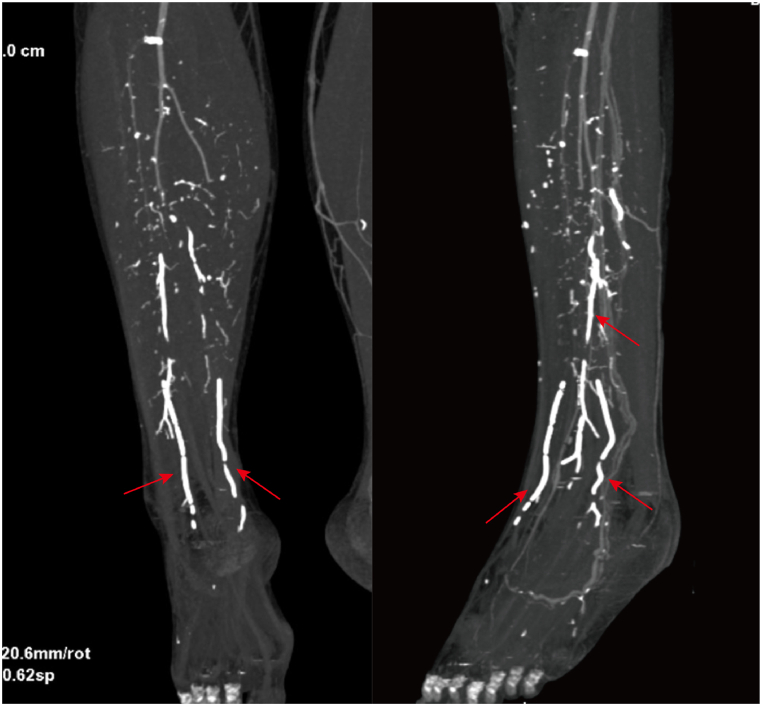
The CT angiography of the arteries of the lower extremities showed a linear, dense signal within the anterior tibial artery, posterior tibial artery, and peroneal artery, which was initially considered to be the results of cement embolization of the arteries (red arrows).

**Fig. 4:**
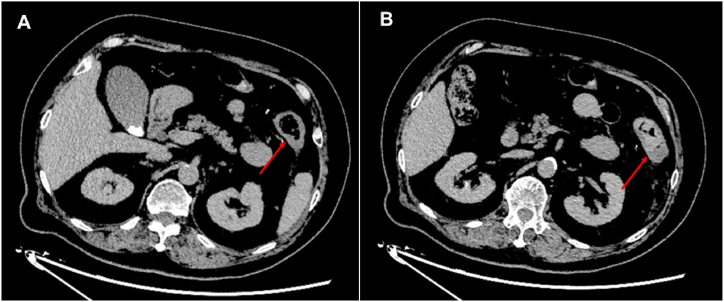
Transverse CT-enhanced images show diffuse bowel wall thickening in the splenic flexure of the colon (A) and bowel wall thickening in the descending colon (B).

**Fig. 5:**
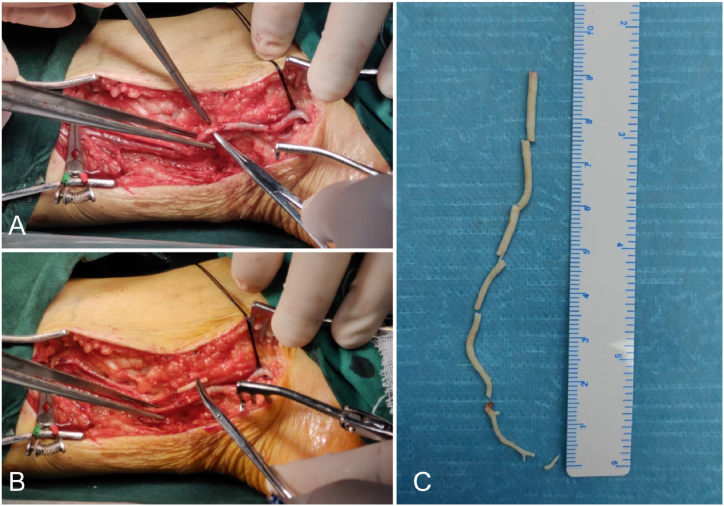
Intraoperative images of embolus removal. (A) Intraoperative picture showing the tubular cemented embolus seen after incision of the left posterior tibial artery, (B) the embolus was clamped using vascular clamps and successfully removed, and (C) the cemented embolus in the posterior tibial artery was removed intact with a length of about 9 cm.

After returning to the ward postoperatively, the patient developed persistent pain in the left lower extremity as well as decreased muscle strength in the left ankle and left toe. Immediate computed tomography (CT) and magnetic resonance imaging (MRI) plain scans of the lumbar spine confirmed that there was no cement leakage into the spinal canal or intervertebral foramina, thus the possibility of neurological complications due to cement leakage was excluded. At about 8 hours postoperatively, the patient experienced a decrease in skin temperature below the level of the left knee. Thus, vascular-related complications were considered possible. Our team immediately consulted with a vascular surgery specialist and then discussed the next steps. Abdominal and lower extremity arteriography confirmed the presence of cement emboli in the inferior mesenteric artery and branches, the right peroneal, posterior tibial, and anterior tibial arteries (Fig. 2, Fig. 3, Fig. 4B). After a few hours, the patient complained of left lower abdominal pain, and abdominal examination revealed pressure and rebound pain in the left lower abdomen. We suspected that this was related to ischemia of the bowel caused by a small embolus in a branch of the inferior mesenteric artery. After consultation with a general surgeon and 48 hours of observation, the patient's abdominal symptoms and signs resolved. After a multidisciplinary consultation, thrombectomy of the posterior tibial artery of the left lower extremity was considered the preferred management. The physician immediately attempted to remove the embolus by incision. After incision of the posterior tibial artery, the cemented embolus was removed from the vessel in segments and the thrombus was removed through a double-lumen embolectomy catheter (Fig. 5A–C). The posterior tibial artery was repaired with an 8-0 PROLENE suture (Ethicon Biosurgery, Somerville, NJ). After suturing the vessel, significant arterial pulsation could be observed.

**Fig. 6:**
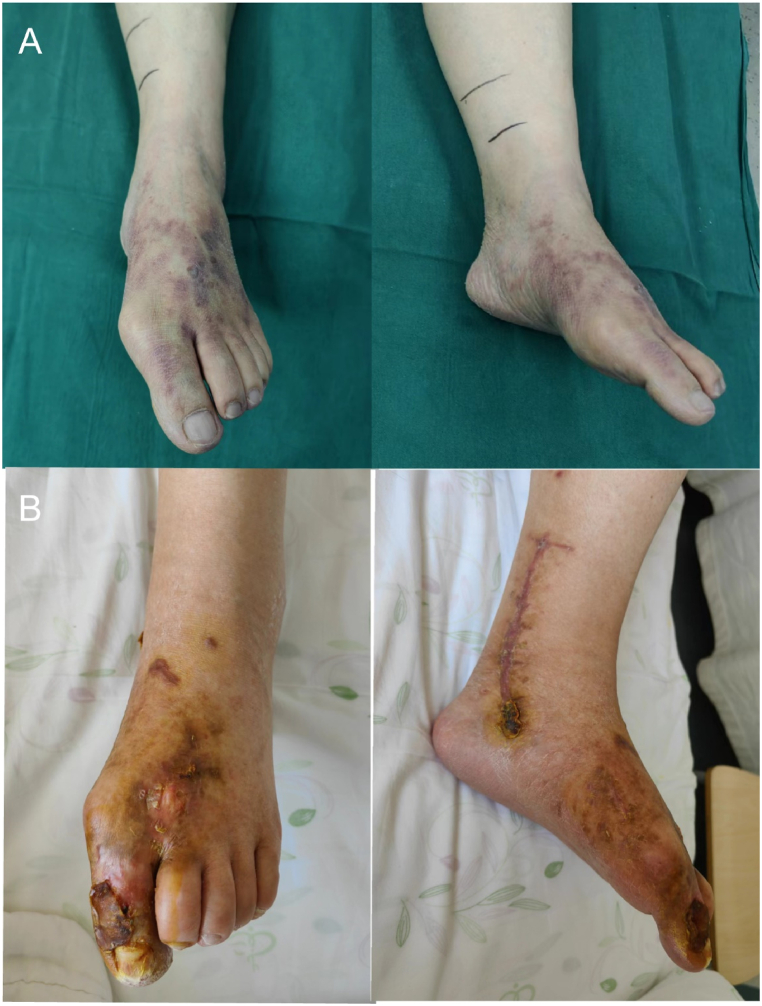
Gross photograph of the left foot of the patient. (A) The preoperative left foot is ischemic and bluish in color, and (B) the left foot at 4 months postoperatively shows partial necrosis of the skin of the distal left thumb without further gangrene.

Postoperatively, the patient did not have any discomfort and had stable vital signs. Postoperative anticoagulation, microcirculatory improvement and decongestion were administered. Around 12 hours postoperatively, the patient's skin temperature at the left heel increased. After 48 hours postoperatively, the patient's skin temperature at the left ankle and left forefoot increased and continued to increase in the subsequent days. Dorsiflexion of the left toe gradually recovered around 3 days postoperatively. At about 2 weeks postoperatively, the patient reverted to transoral feeding and did not complain of any abdominal discomfort. At about 3 weeks postoperatively, the sutures were removed from the left calf wound, which was healing well. At the 3-month follow-up, the patient's skin temperature in the left lower thigh was almost normalized (Fig. 6A–B), with significant recovery of sensation and movement, and she could walk on the ground with the assistance of a walker.

## 3 Discussion

Percutaneous vertebral augmentation techniques have been widely utilized for the management of osteoporotic and traumatic compression fractures, pathological fractures secondary to vertebral metastases, and pedicle screw augmentation. Reported procedural complications are predominantly related to cement leakage, such as extravasation into the paravertebral region, subcutaneous tissues, and the venous or arterial system. However, cases of bone cement leakage into the peripheral arteries resulting in severe symptoms are exceedingly rare. This case represents the only documented case to date of bone cement extravasation into a peripheral artery causing severe symptoms, which was successfully managed through surgical intervention.

Previous studies have reported a variety of vascular complications, most of which were due to embolization of the cardiopulmonary veins caused by cement entering the vena cava through the periportal vein (Table 1) [7,10]. Because the pressure in the periarterial arteries is greater than in the veins, extravasated bone cement rarely enters the arteries. However, embolization of the arteries often leads to serious consequences. A study by Amoretti et al. [8] reported the case of a 72-year-old woman who developed extravasation of bone cement into the abdominal aorta after undergoing percutaneous vertebroplasty for bone metastases from breast cancer. Fortunately, the patient was asymptomatic and had no other consequences during a four-month follow-up. Mozaffar et al. [9] reported a rare and serious complication of cement extravasation through the abdominal aorta into the peripheral arteries after percutaneous vertebroplasty for pathologic fractures of multiple vertebrae. Color doppler ultrasound demonstrated embolization of the anterior and posterior tibial arteries in both lower extremities. After 18 hours of confirmed embolization, this patient was admitted to the operating room for interventional thrombectomy with a thrombolytic catheter via the femoral artery. However, postoperative imaging showed that the cemented embolus in the anterior and posterior tibial arteries was not completely retrieved. The patient developed a postoperative complication of suspected infectious shock and died on the third postoperative day. We consider that they failed to completely retrieve the embolus and promptly restore blood flow to the lower extremities contributing to the adverse outcome of the patient. Meanwhile, the large intraoperative injuries exacerbated the mortality of the patient. Consistent with findings from previous related studies, the present case also exhibited signs of ischemia in the arterial supply region during the early postoperative period, typically presenting as limb numbness, pain, and functional impairment. These observations suggest that similar postoperative symptoms should raise suspicion for the possibility of arterial embolism.

**Table 1:** The literature review of artery bone cement embolization caused by PVP.

Author	Time of embolization detected	Clinical present	Features of the cement embolus (size/shape/localization)	Treatment strategies	Outcome
Ku et al.	2 years postoperatively	Dyspnoea and chest tightness.	Multiple branching and high-density tubular opacities in three segmental pulmonary arteries, linear shape of the cement leakage from within the vertebral body, proceeding into the perivertebral venous system.	Conservative treatment	Symptoms relieved
Amoretti et al.	During the procedure	Asymptomatic	Hook-shaped cement fragment in the aorta.	Conservative treatment	Discharged
Sidhu et al.	3 hours postoperatively	Non-colicky mid-abdominal and back pain.	Diffuse cement embolism in both kidneys, inferior mesenteric artery and its branches, linear shape of the cement into the aorta.	Conservative treatment	Discharged
Mozaffar et al.	12 hours postoperatively	Pain and numbness in both lower limbs.	Linear shape of the cement in posterior and anterior tibialis arteries.	Embolectomy	Dead
Panagiotis et al.	5 hours postoperatively	Weakness of foot extensors, coldness of medial edge of left foot.	Linear shape of the cement in dorsal foot artery.	Conservative treatment	Discharged and recovered after 6 weeks
Our study	8 hours postoperatively	Weakness in foot flexion and extension. Pain, coldness, numbness below the middle of the shins.	Linear shape of the cement in posterior tibial, anterior tibial and peroneal arteries.	Embolectomy	Discharged

As seen on the postoperative CT scan of the lumbar spine, the cement entered the abdominal aorta through the lumbar artery at the L3 level (Fig. 3). Some of the cement was flushed into the lower limbs with the blood flow in the abdominal aorta, and the other part of the cement crossed the abdominal aorta into the inferior mesenteric artery at the same level, which ultimately caused embolization of the inferior mesenteric and lower limb arteries. We consider the abundant and likely variations of nutrient blood vessels around the vertebral body invaded by the tumor in this patient to be an important factor leading to serious complications. Previous studies have reported that the amount of bone cement injected correlates with cement leakage [11,12]. If too much bone cement is injected, the space within the vertebrae narrows, leading to increased pressure within the vertebral body, which enables excess cement to overflow into the blood vessels, especially the venous system. However, in our case, cement leakage at the level of the L3 vertebral body occurred at the initial stage of injection and no substantial amount of cement was injected in that vertebral body. It is very rare that most of the bone cement enters directly into the arterial system through the vertebrae narrows at the initial stage of injection. That also reminds us that it is very important to timely observe the visualization of the cement in the vertebral body after injection of the cement. If the amount of cement in the vertebral body does not match the amount injected, the injection should be promptly stopped, and the reason should be examined.

In addition, the viscosity of the bone cement is recognized as being closely related to cement leakage. Generally, a viscous, toothpaste-like bone cement that undergoes an adequate polymerization reaction is recommended [13,14]. Several studies have proposed a staged strategy for injecting bone cement [15,16]. A small amount of bone cement is injected at the beginning of the injection to occlude the endangered vessels, and the remaining bone cement is further injected after confirming that no leakage is detected. At the same time, the speed and strength of injection need to be strictly controlled. When resistance to injection is encountered, the cause should be examined immediately to confirm that there is no inflow of bone cement into the vessel before continuing the injection.

Some authors recommend preoperative or intraoperative intervertebral angiography for early evaluation of peri-fracture blood supply and prevention of possible vascular-related complications [17]. However, it seems difficult to widely implement this procedure in all patients with spinal fractures since it would prolong hospitalization and increase the cost of medical care. We suggest that preoperative angiography is necessary for high-risk patients, especially those with pathologic vertebral fractures caused by metastases from malignant tumors, and that it will help to reduce the occurrence of serious complications.

## 4 Conclusion

Surgeons should be more cognizant of the potential risk of arterial embolism due to bone cement leakage during PVP, despite its rarity. The rare case we report here contributes valuable insights into the early identification and management of this complication. Awareness of this risk is particularly critical when treating high-risk patients. Vascular complications should be considered in the differential diagnosis for patients presenting with postoperative lower extremity pain and numbness. In terms of therapeutic strategies, arterial embolectomy may be a viable option for managing such complications. However, the decision to proceed with surgery should be made cautiously, based on a thorough evaluation of the patient's condition and the timing of intervention.

## CRediT authorship contribution statement

**Jiashen Shao:** Writing – original draft, Investigation, Data curation, Conceptualization. **Hai Feng:** Methodology, Investigation, Data curation. **Bin Liu:** Methodology, Investigation. **Hai Meng:** Supervision, Resources, Project administration, Conceptualization. **Shili Ning:** Supervision, Resources, Project administration. **Yingchi Yang:** Validation, Supervision. **Yun Yang:** Validation, Supervision. **Xuehu Xie:** Validation, Resources. **Zihan Fan:** Validation, Supervision. **Zhiwu Zhang:** Validation, Resources. **Nan Su:** Visualization, Validation. **Jinjun Li:** Writing – review & editing, Validation, Supervision. **Qi Fei:** Writing – review & editing, Visualization, Validation, Supervision, Resources, Data curation, Conceptualization.

## Ethical approval

Since this study belonged to a single case report, the data were anonymous, and had no identifiable information about the patient, the need for ethics approval was waived by Ethics Committee of the Beijing Friendship Hospital.

## Consent for publication

Written informed consent was obtained from the patient for publication of this case report and any accompanying images.

## Data availability statement

The original contributions presented in the study have been included in the article. According to the requirements of the patient, further data will be made available from the corresponding author on reasonable request.

## Funding

Supported by Beijing Friendship Hospital, Capital Medical University (No. YYZZ202230).

## Declaration of competing interest

The authors declare that they have no known competing financial interests or personal relationships that could have appeared to influence the work reported in this paper.
